# Trends in genetic patent applications: the commercialization of academic intellectual property

**DOI:** 10.1038/ejhg.2013.305

**Published:** 2014-01-22

**Authors:** Jannigje G Kers, Elco Van Burg, Tom Stoop, Martina C Cornel

**Affiliations:** 1 Faculty of Earth and Life Sciences, VU University Amsterdam, The Netherlands; 2 Community Genetics, Department of Clinical Genetics/EMGO Institute for Health and Care Research, VU University Medical Center, Amsterdam, The Netherlands; 3 Faculty of Economics and Business Administration, VU University Amsterdam, The Netherlands; 4 The Netherlands Patent Office, NL Agency, Ministry of Economic Affairs, The Hague, The Netherlands; 5 Center for Society and the Life Sciences, Nijmegen, The Netherlands

**Keywords:** patent application trends, genetics, academic entrepreneurship, intellectual property

## Abstract

We studied trends in genetic patent applications in order to identify the trends in the commercialization of research findings in genetics. To define genetic patent applications, the European version (ECLA) of the International Patent Classification (IPC) codes was used. Genetic patent applications data from the PATSTAT database from 1990 until 2009 were analyzed for time trends and regional distribution. Overall, the number of patent applications has been growing. In 2009, 152 000 patent applications were submitted under the Patent Cooperation Treaty (PCT) and within the EP (European Patent) system of the European Patent Office (EPO). The number of genetic patent applications increased until a peak was reached in the year 2000, with >8000 applications, after which it declined by almost 50%. Continents show different patterns over time, with the global peak in 2000 mainly explained by the USA and Europe, while Asia shows a stable number of >1000 per year. Nine countries together account for 98.9% of the total number of genetic patent applications. In The Netherlands, 26.7% of the genetic patent applications originate from public research institutions. After the year 2000, the number of genetic patent applications dropped significantly. Academic leadership and policy as well as patent regulations seem to have an important role in the trend differences. The ongoing investment in genetic research in the past decade is not reflected by an increase of patent applications.

## INTRODUCTION

For many years, protecting and commercializing intellectual property (IP) through patents on human materials like polypeptides, genes, nucleotides and living organisms has been a point for debate.^1, 2, 3, 4, 5^ Patents may have a role in the translation of genetic research in commercial applications.^6^ Some researchers argue that protecting a discovery is essential to increase innovation activities and also to increase the chances that investments in research and development will be profitable.^7^ Protecting a technological innovation is done by applying for a patent on the invention. A patent forms an IP right that gives the owner the exclusive rights to the commercial use of a technical invention for a maximum of 20 years.^1, 8^

Information about patent applications can be used to derive trends in research areas and to identify new developments at an early stage.^9^ Today, patent data have become more readily available for research purposes due to the increase in searchable public online databases,^10^ thereby enabling trends in genetic patent applications (GPAs) to be analyzed. As patents give an owner the right to exclude others from using the invention, they are often considered as a key indicator for research and innovation output.^11, 12, 13^ Patented inventions are often licensed to established companies and venture-capital-backed start-ups that create jobs for highly educated scientists.^14^ However, focusing on patents in isolation may misrepresent the nature of the impact of universities on the economy.^15^ The growing number of patent applications by universities indicates that efforts to commercialize university inventions have increased dramatically.^3^ Previous research showed that university patent activities were often related to commercialized outputs and that universities, for instance, occupy leading roles in the development of important pharmaceuticals.^2, 16, 17^

The commercialization of IP, indicated by patent-related activities, is controversial in genetics due to the possible negative effects of patenting human material, for instance, on the availability of diagnostics and treatments. Some critics argue that biological material by definition cannot be patented, because it has always existed in nature and thus does not constitute an invention but rather a mere discovery.^1^ Other scholars argue that the technology would not have developed if discoveries had not been commercially protected, and neither would the field of genetic research have increased.^18^ Additionally, without patents, the increase in knowledge might have been slower^19^ Despite this debate, a substantial proportion of the human genome has already been patented by drug and biotech firms and research institutions,^20, 21^ thereby raising discussions on the impact of patents for developing medicines, as researchers may be constrained in their activities. A famous case is the commercial company Myriad, which has the rights to commercialize specific hereditary breast cancer genes.^22^ For several years now, people have objected to these patents due to the potential impact on diagnostic testing.^23, 24^ However, in June 2013, the Supreme Court decided in the Myriad case that human genes, defined as genomic DNA, are not patentable.^25^, ^26^, ^27^

To better understand the trends in GPAs, we studied by whom, when and where patent applications were filed. We used the International Patent Classification (IPC) to collect patent applications filed with the World Intellectual Property Organization (WIPO) and the European Patent Office (EPO), using the European version of the patent classification (ECLA). Previous research observed growth in US-based genetic patents and GPAs up to the year 2000 and provided various explanations for this pattern. These explanations include the end of the human genome project and unprecedented growth in large-scale sequencing around 2000^22, 28^ and new guidelines within the US Patent and Trademark Office (USPTO).^5^ We studied time trends between 1990 and 2009 and explored geographic distribution trends on four different levels: international, per continent, per country, and per university in one country (The Netherlands). In this period, much human and financial investments were made in genetic research.^28^, ^29^, ^30^

## MATERIALS AND METHODS

### Patent selection

GPAs are those patent applications containing at least one genetic-based term, for example, nucleic acids, genetic material, mutation or genetic engineering (as specified in the supplementary data). This means that we include not only human GPAs but also genetic modifications in plants, fungi, bacteria or animals. The patent applications that apply are EP (an application filed at the EPO) and PCT applications (an application filed at WIPO under the Patent Cooperation Treaty (PCT)). A benefit of using EP and PCT patent application data is the possibility to compare data across countries, as other metrics often lack this ability of international comparison. The study's analysis was done by using the European version (ECLA) of the IPC and choosing specific classification numbers with genetic terms in the description, for example: ‘C12N 1/11; modified by introduction of foreign genetic material' and ‘A61K 31/712; nucleic acids or oligonucleotides having modified sugars, that is, other than ribose or 2′-deoxyribose' (Source: WIPO Statistics Database, August 2011). We did not use additional keywords. The entire search algorithm and the list of used patent classifications are attached as Supplementary Information. In the ECLA and IPC code system, as older patents are updated to reflect newer classifications, reclassifications in the ECLA or IPC system do not affect our analysis.

The time period investigated ranged from 1990 until 2009. The data collection was carried out in PATSTAT – also known as the EPO Worldwide Statistical Database – using the selected ECLA codes. The patent application data collected in PATSTAT consisted of published patent application families, where a patent application family is a set of patent applications from various countries that originate from the same first filing and usually protect a single invention.^23^ Our study thus considers published ‘international' patent applications, which consist of the sum of the PCT- and EP-published patent application families. A limitation of using EP and PCT patent applications is that this excludes published national and regional patent applications and patent applications that are not published. As a consequence, patent families that do not include published PCT or EP patent applications are not covered. The geographic distribution of GPAs was studied on the international level, by continent of the applicant, by country of the applicant, and among different universities. To explore the differences between universities at a national level, The Netherlands was selected as a typical country.

To analyze the data, the following software and databases were used: Patent Database Espacenet, Epoline patent register, Epoque database, PATSTAT, National Centre of Biotechnology Information (NCBI), Pubmed, and Google Scholar. We tested the data using a multiple linear regression.

## RESULTS

**Figure 1:**
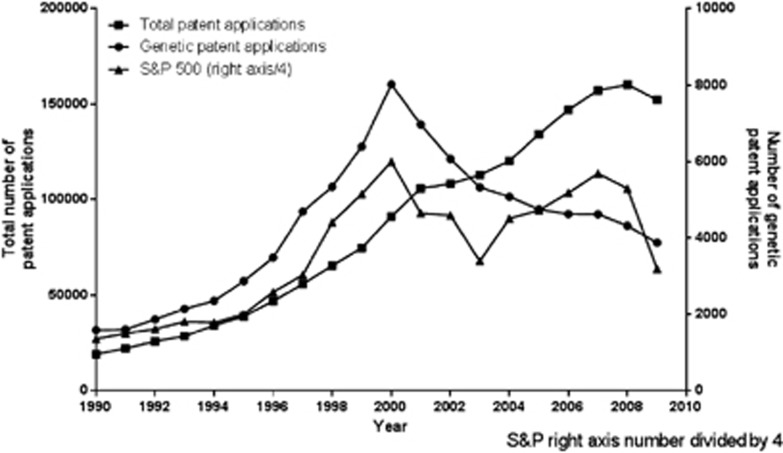
Total number of patent applications, genetic patent applications and S&P 500 values from 1990 to 2009.

**Figure 2:**
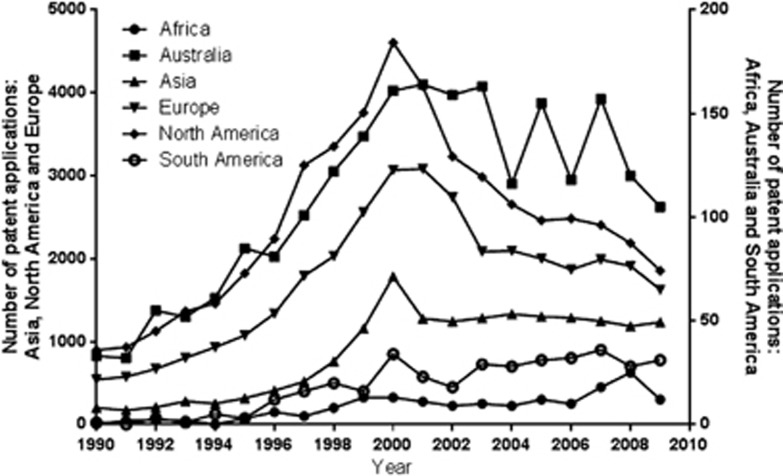
Trends in GPAs per continent.

**Figure 3:**
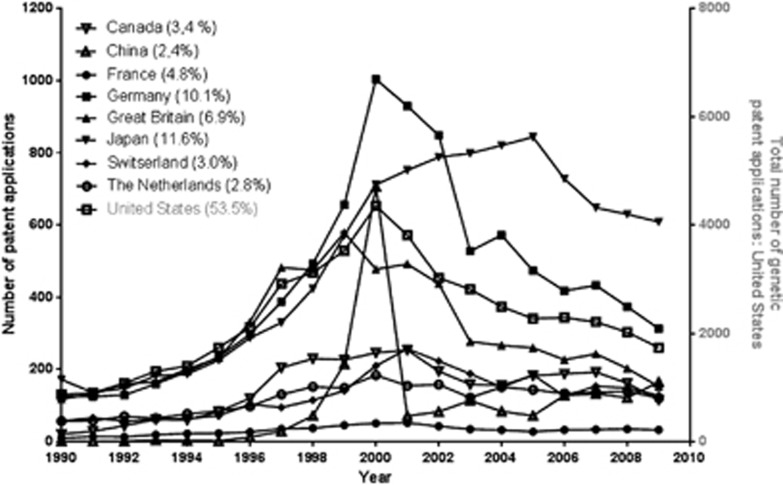
Trends in GPAs in nine countries.

**Figure 4:**
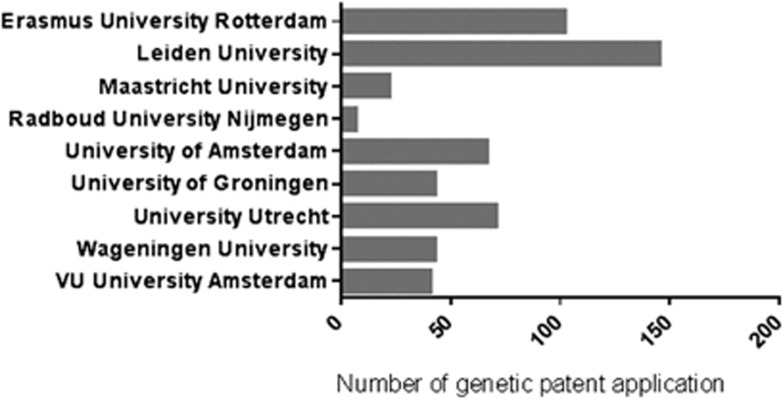
Total number of GPAs by Dutch Universities, 1990–2009.

The GPAs on the international level, in different continents, countries, and universities were explored by using PATSTAT and the Espacenet worldwide database between 1990 and 2009, covering patent applications filed within the PCT and EP systems (patent applications that are only filed nationally are not included). Key findings from the longitudinal analysis of GPAs between 1990 and 2009 are shown in Figures 1, 2, 3, 4.

In the period from 1990 until 2009, the number of patent applications filed internationally increased (Figure 1). The filing of international patent applications increased in a linear fashion until in 2009 around 152 000 patent applications were filed. GPAs have increased as well, although not linearly. As can be seen in Figure 1, there was a peak in GPAs in 2000. From 1990 to 2009, the international growth rate of GPAs is about 276%, whereas in the same period the total patent application growth rate is 794%. The S&P 500 stock market index, indicating the economic climate, showed the same pattern as the GPAs until 2003.

To analyze continent-specific patterns, Figure 2 shows the number of GPAs per continent. Compared with 2000, North America and Europe experienced a decrease in GPAs of 50%. Although the totals are relatively low compared with North America and Europe, it is still interesting to note that after 2000 an increase can be seen for the continents of South America and Africa, while in Asia the annual number after 2000 is more or less stable (>1000 per year) and Australia (100–170 per year). Continental differences are observed, which might be explained by the growing economies in non-Western countries and changes in legislation and procedures like the European regulation on legal protection of biotechnological inventions.

Figure 3 shows country-specific patterns of nine countries, which together account for 98.9% of the total number of GPAs between 1990 and 2009. The United States is responsible for 53.3% of the GPAs filed internationally within the EP and PCT systems and China for 2.4%. Most countries, including the United States and China, show a peak at or around the year 2000. In the case of China, this increase in 2000 was due to two companies and might be related to political change and economic development. Biowindow Gene Development Inc., Shanghai, China filed 22 applications in 1990, 260 applications in 2000, and 1 application in 2001. Biodoor Gene Technology Ltd., Shanghai, China has no applications in 1990, 59 in 2000, and no applications in 2001.

The classification of the patent applications made it possible to identify broader technological areas in which GPAs are applied for, to explore whether or not there was any variation over time in different technological areas to explain the peak in 2000. Distinguishing five technology areas (Biotechnology 65%, Pharmaceuticals 18.5%, Measurement 7.1%, Organic fine chemistry 3.5%, and Food chemistry 1.7%, which account for >95% of all GPAs) showed similar patterns in different technology areas (data not shown).

Our results at the national level, focusing on The Netherlands, show important differences between research institutions. The Netherlands has approximately 2400 international GPAs in the period between 1990 and 2009. The entities with >5 GPAs were divided between private/industry and public research institutions (government, universities, research institutes, and hospitals). Private institutions applied for 73.3% of the GPAs, and public institutions are responsible for 26.7% of the GPA. There was a significant increase in the number of GPAs by the Dutch universities between 1990 and 2009 (multiple regression results: *P*=0.000, *F*=44.834, R2=0.714). A peak can also be seen in The Netherlands in 2000. Compared with 2000, there was a decrease of 38.6% in GPAs in 2001. Figure 4 shows the total number of GPAs, which shows large differences between Dutch universities. The numbers of GPAs are specified for the Dutch universities with >5 GPAs, which is why the three Dutch technology universities are not shown in the overview. With regard to the Dutch public institutions, 85.5% of the public GPAs originate from the Dutch universities.

## DISCUSSION

The aim of this paper was to provide insights into the trends of GPAs, with a focus on patent application trends between 1990 and 2009 on the international level, per continent, per country, and at Dutch universities. In comparing the growth in GPAs to the overall growth of patent applications, genetic patenting showed an unparalleled growth till 2000 (Figure 1). The literature provides different explanations for the increase of patenting activities in biosciences and engineering. The first explanation for the increase in the number of GPAs until 2000 can be found in the investments related to the sequencing of the human genome as well as the large perceived market opportunities for some bioscience engineering products.^28, 31^ The second explanation might be the financial support provided by the biotechnology and pharmaceuticals industries, which led to a direct increase in commercialization outcomes, with an incidental effect of the possibility to patent genomic-based inventions.^28, 29^

Since 1980, it has been allowed to patent life forms in the US, and patenting inventions that used life forms was already possible long before then. Shortly after many patents applications on genetics were filed, an influential public debate ensued and several governments (eg, the Bush Administration) also took action to influence the direction of genetic research activities.^22^ Soini *et al*^5^ engaged in a dialogue on patenting and licensing in genetic testing, including ethical, legal, and social issues, and summarized developments in the previous decades. James Watson argued: ‘human genes are unique and convey information about the essences of being human, they should not be patented, the human genome project was intended to benefit all, not just select companies, and finally, patents on human genes are not necessary, but if the are granted, compulsory licenses should be required to ensure fair access'.^32^ The European ‘directive on the legal protection of biotechnological inventions' (EU/98/44), published in 1998,^8^ stipulated that the simple discovery of the sequence or partial sequence of a gene is not considered patentable. However, an element isolated from the human body or produced by means of a technical process, including the sequence or partial sequence of a gene, may constitute a patentable invention. Moreover, in 2001 an important adjustment was made to patent regulations in the US, which stated that patent applications for DNA sequences had to explain the function of the DNA sequence that was to be patented (USPTO).

To explain the decrease in GPAs after 2000, Lawrence^33^ pointed to the fact that the Human Genome Project was completed. The decrease in GPAs took place after the increase of concerns voiced in the public debate, for instance, in the United States in 2001 when the New York Times wrote about patents on genetically engineered seeds and initiated a public discussion on genetic patenting.^34^ The discussion about genetic patents and GPAs continues in the literature.^5, 22, 35^ The patentability of a DNA sequence, individual mutations, SNPs, and gene variants became controversial, as opposed to patents on a diagnostic test or therapeutic protein.^5^

The United States is responsible for the greater part of the 2000 peak, as more than half of the international GPAs originate from there. However, similar patterns were also found in Europe. An explanation for continental differences may be the difference in patent strategies of applicants or patent examiners. Different examination approaches of the patent offices in Europe and the United States could explain the current legal status of some genetic diagnostic-testing patent applications.^22, 23^ An additional explanation may be the differences in economies, like the increased investments in genomics in the nineties and the possibility to patent genes that made it more interesting for the industry to invest in genomics. Moreover, Asia and South America are rapidly developing economies, while in the western economies after 2000 many investors lost their confidence in the biotech sector (see in Figure 1 the identical patterns of the S&P500 and the GPAs), which in turn can explain a decrease in international patent applications and GPAs.

At the country level, just nine countries are responsible for 98.9% of international GPA filings (Figure 3). Important differences are observed between these nine countries, which likely are related to differences in laws and economic variation. The data of nine international patent applications originating from China shows that a few individual applicants can create a large shift in the country-level patterns.

Regarding the national level, Cook-Deegan and Heaney^22^ observe that in the United States 39% of the granted DNA patents are owned by academic institutions (in the period 1980–1993), which is relatively high, as for the total of patents the percentage from academic institutions is only 5%. This might partly be explained by the fact that genetic research was relatively important at universities, compared with older technologies. Biotech researchers in universities found that unlike researchers in other sciences their research results had the potential to be both patentable and profitable. Universities with such departments therefore became active in filing patent applications. Universities were encouraged to set up TTOs and file applications, for instance, by governments, governing bodies, and law changes. In The Netherlands, 26.7% of the international GPAs are from the public research institutions, 85.5% of which are from universities. Large differences in the number of GPAs exist among Dutch universities (Figure 4). Leiden University would appear to be leading in patent applications, with especially the Clinical Genetics Centre and their head of the Department of Human Genetics (Professor GB van Ommen) filing many patent applications. Researchers who are the head of a department in combination with top research positions appear to have the most patent applications to their name in the field of genetics. Other examples of such researchers are Rene Bernards from The Netherlands Cancer Institute (NKI), Frank Grosveld, Chair of Cell Biology at the Erasmus University Rotterdam, and Mathieu Noteborn, Professor in Molecular Genetics at Leiden University. The patenting activity of one single professor and one research centre are thus the main driving forces behind the observed pattern. These results are consistent with the role of leadership described in a study by Bercovitz and Feldman,^36^ which explains that the chair of the department has an influence on the commercialization of IP. The chair's activities will have an effect on other members of the department, stimulating them to become more involved in patenting as well.^36, 37^ Thus, the observed differences between universities might be explained on a micro level by differences among research group leaders within universities as well as by differences in technology transfer-support infrastructures.^31, 38^

Three studies described trends in genetic patents in the past, using a method different from ours, for example, by looking at published patents instead of published patent applications; results from these studies show similarities as well as differences when compared with our findings.^22, 39, 40^ Oldman^40^ searched for trends in patent claims in genomics, proteomics, and biotechnology by using keywords in the Espacenet titles such as genes, proteins, DNA, amino acids, nuclide nucleic acids, and RNA in the time period 1990–2003. Unfortunately, as this study used a different method the results are difficult to compare. Nevertheless, the result showed a peak in published patents around 2002, which indicates a peak in patent applications in 2000. Cook-Deegan and Heaney^22^ investigated granted US patents using the Delphion Patent Database based on the USPTO classification system and considered the time frame 1984–2008. The analysis of US DNA patents by Cook-Deegan and Heaney^22^ also shows a peak, although it is less pronounced and somewhat later, around 2001. A possible explanation for the different pattern is that Cook-Deegan and Heaney^22^ use a different database and an algorithm that searches for granted US patents rather than published patent applications, which appears to result in a different trend, as it is difficult to compare patent applications with granted patents. Oldman and Hall^39^ also investigated patent applications, using the same method as our study (PATSTAT), which resulted in a similar trend in total patent applications. However, they used different genetic/biotech IPC codes (including biotechnology in a broad sense), which resulted in GPA figures that could not be compared.

### Limitations

This study is not without its limitations. First of all, the register of patent data unavoidably contains some errors, such as mis-spellings of author or institution names (eg, verengning vu-windesheim). Second, the choice by a patent examiner of a specific IPC number defines whether a patent application is considered as a genetic patent application or not, while this category might also include patent applications that have barely any genetic content and could also exclude patent applications that may be related to genetics. Third, our choice of IPC numbers defines which patent applications we consider as being related to genetics. Others might choose a more restricted selection, as applications for a patent on a gene are just a fraction of the applications that were revealed in our search. Fourth, some patent applications have more than one IPC number, including only one of a few genetic code numbers, which implies that sometimes other characteristics than ‘genetic' might characterize the application better. Therefore, some patent applications might be less related to genetics than that appears from the IPC numbers. These limitations may influence the results. Finally, this study focused on patent applications within the international EP and PCT systems to enable meaningful comparisons between countries and continents. Also including national-only filings as well might generate different patterns, in particular with regard to the United States and China.

## CONCLUSIONS

Since 1990, the number of international GPAs increased up to 2000 and declined by 50% thereafter. The peak is seen in the United States and Europe, while Asia shows a stable level, and Africa and Australia show a small but gradual increase. Nine countries together account for 98.9% of GPAs. Public institutions and universities have applied for relatively many genetic patents. One or several leaders within an institution can strongly influence trends in GPAs. Finally, the ongoing investments in genetic research after 2000 are not reflected in an increase in the number of GPAs.
