# Supplementary material for: Trends in genetic patent applications: the commercialization of academic intellectual property
Source: Eur J Hum Genet. 2014 Jan 22;22(10):1155–9. doi: 10.1038/ejhg.2013.305 (PMC4169532; doi:10.1038/ejhg.2013.305)
Supplement: Supplementary Information [file ejhg2013305x1.doc]

**SUPPLEMENTARY INFORMATION**

**PATSTAT Search Algorithm**

The following algorithm is used in PATSTAT to gather the patent information:

(A01H1/00 or A01H1/06 or A01H4/00 or A01K67/00 or A61K48/00 or A61K31/7088 or

A61K31/7105 or A61K31/711 or A61K31/7115 or A61K31/712 or A61K31/7125 or

A61K31/713 or C12N1/11 or C12N1/13 or C12N1/15 or C12N1/19 or C12N1/21 or

C12N5/10 or C12N7/01 or C12Q1/68 or C07H21/00 or C07H21/02 or C07H21/04 or

C12P19/30 or C12P19/32 or C12N15*)/ic/ec AND (EP or WO)/pn AND OPD >= '1990'

AND OPD <= '2010'

**Patent Classifications**

IPC8 -Technology Concordance. Source: WIPO Statistics Database. Last update: August

2011

AGRICULTURE

A01H 1/00 Processes for modifying genotypes (A01H 4/00 takes precedence) [5]

A01H 1/06 Processes for producing mutations, e.g. treatment with chemicals or with

radiation (specific mutations prepared by genetic engineering on plant cell or

plant tissues C12N 15/00)

A01H 4/00 Plant reproduction by tissue culture techniques [5]

A01K 67/00 Rearing or breeding animals, not otherwise provided for; New breeds of

animals (methods for reproduction or fertilisation A61D 19/00; medicinal

preparations containing sperm A61K 35/52; tissue- or animal-cell cultivation

apparatus C12M 3/00; cultivation or maintenance of tissue or animal cells

C12N 5/00; mutation or genetic engineering C12N 15/00)

MEDICAL OR VETERINARY SCIENCE; HYGIENE

A61K 31/7088 Compounds having three or more nucleosides or nucleotides [7]

A61K 31/7105 Natural ribonucleic acids, i.e. containing only riboses attached to adenine,

guanine, cytosine or uracil and having 3'-5' phosphodiester links [7]

A61K 31/711 Natural deoxyribonucleic acids, i.e. containing only 2'-deoxyriboses attached

to adenine, guanine, cytosine or thymine and having 3'-5' phosphodiester links

[7]

A61K 31/7115 Nucleic acids or oligonucleotides having modified bases, i.e. other than

adenine, guanine, cytosine, uracil or thymine [7]

A61K 31/712 Nucleic acids or oligonucleotides having modified sugars, i.e. other than ribose

or 2'-deoxyribose [7]

A61K 31/7125 Nucleic acids or oligonucleotides having modified internucleoside linkage, i.e.

other than 3'-5' phosphodiesters [7]

A61K 31/713 Double-stranded nucleic acids or oligonucleotides [7]

A61K 48/00 Medicinal preparations containing genetic material which is inserted into cells

of the living body to treat genetic diseases; Gene therapy [5]

ORGANIC CHEMISTRY

C07H 21/00 Compounds containing two or more mononucleotide units having separate

phosphate or polyphosphate groups linked by saccharide radicals of nucleoside

groups, e.g. nucleic acids [2]

C07H 21/02 with ribosyl as saccharide radical [2]

C07H 21/04 with deoxyribosyl as saccharide radical [2]

C12N 1/11 modified by introduction of foreign genetic material [5]

C12N 1/13 modified by introduction of foreign genetic material [5]

C12N 1/15 modified by introduction of foreign genetic material [5]

C12N 1/19 modified by introduction of foreign genetic material [5]

C12N 1/21 modified by introduction of foreign genetic material [5]

C12N 5/10 Cells modified by introduction of foreign genetic material, e.g. virustransformed

cells

C12N 7/01 Viruses, e.g. bacteriophages, modified by introduction of foreign genetic

material (vectors C12N 15/00)

C12N 15/00 Mutation or genetic engineering; DNA or RNA concerning genetic

engineering, vectors, e.g. plasmids, or their isolation, preparation or

purification; Use of hosts therefor (mutants or genetically-engineered microorganisms

C12N 1/00, C12N 5/00, C12N 7/00; new plants A01H; plant

reproduction by tissue culture techniques A01H 4/00; new animals A01K

67/00; use of medicinal preparations containing genetic material which is

inserted into cells of the living body to treat genetic diseases, gene therapy

C12P19/30· · · Nucleotides [3]

[C12P 19/32](http://web2.wipo.int/ipcpub/) · · · · having a condensed ring system containing a six-membered ring having two nitrogen atoms in the same-ring, e.g. purine nucleotides, nicotineamide-adenine dinucleotide [3]

A61K 48/00; peptides in general C07K) [6]

*Note(s) - This group covers processes wherein there is a modification of the*

*genetic material which would not normally occur in nature without*

*intervention of man which produce a change in the gene structure which is*

*passed on to succeeding generations.*

C12N 15/01 · Preparation of mutants without inserting foreign genetic material therein;

Screening processes therefor [5]

C12N 15/02 · Preparation of hybrid cells by fusion of two or more cells, e.g. protoplast

fusion [5]

C12N 15/03 · · Bacteria [5]

C12N 15/04 · · Fungi [5]

C12N 15/05 · · Plant cells [5]

C12N 15/06 · · Animal cells [5]

C12N 15/07 · · Human cells [5]

C12N 15/08 · · Cells resulting from interspecies fusion [5]

C12N 15/09 · Recombinant DNA-technology [5]

C12N 15/10 · · Processes for the isolation, preparation or purification of DNA or RNA

(chemical preparation of DNA or RNA C07H 21/00; preparation of nonstructural

polynucleotides from micro-organisms or with enzymes C12P 19/34)

[5]

C12N 15/11 · · DNA or RNA fragments; Modified forms thereof (DNA or RNA not used in

recombinant technology C07H 21/00) [5]

C12N 15/113 · · · Non-coding nucleic acids modulating the expression of genes, e.g.

antisense oligonucleotides

C12N 15/115 · · · Aptamers, i.e. nucleic acids binding a target molecule specifically and with

high affinity without hybridising therewith

C12N 15/117 · · · Nucleic acids having immunomodulatory properties, e.g. containing CpGmotifs

C12N 15/12 · · · Genes encoding animal proteins [5]

C12N 15/13 · · · · Immunoglobulins [5]

C12N 15/14 · · · · Human serum albumins [5]

C12N 15/15 · · · · Protease inhibitors, e.g. antithrombin, antitrypsin, hirudin [5]

C12N 15/16 · · · · Hormones [5]

C12N 15/17 · · · · · Insulins [5]

C12N 15/18 · · · · · Growth hormones [5]

C12N 15/19 · · · · Interferons; Lymphokines; Cytokines [5]

C12N 15/20 · · · · · Interferons [5]

C12N 15/21 · · · · · · Alpha-interferons [5]

C12N 15/22 · · · · · · Beta-interferons [5]

C12N 15/23 · · · · · · Gamma-interferons [5]

C12N 15/24 · · · · · Interleukins [5]

C12N 15/25 · · · · · · Interleukin-1 [5]

C12N 15/26 · · · · · · Interleukin-2 [5]

C12N 15/27 · · · · · Colony stimulating factors [5]

C12N 15/28 · · · · · Tumor necrosis factors [5]

C12N 15/29 · · · Genes encoding plant proteins, e.g. thaumatin [5]

C12N 15/30 · · · Genes encoding protozoal proteins, e.g. from Plasmodium, Trypanosoma,

Eimeria [5]

C12N 15/31 · · · Genes encoding microbial proteins, e.g. enterotoxins [5]

C12N 15/32 · · · · Bacillus crystal proteins [5]

C12N 15/33 · · · · Genes encoding viral proteins [5]

C12N 15/34 · · · · · Proteins from DNA viruses [5]

C12N 15/35 · · · · · · Parvoviridae, e.g. feline panleukopenia virus, human parvovirus [5]

C12N 15/36 · · · · · · Hepadnaviridae [5]

C12N 15/37 · · · · · · Papovaviridae, e.g. papillomaviruses, polyomavirus, SV40 [5]

C12N 15/38 · · · · · · Herpetoviridae, e.g. herpes simplex virus, varicella-zoster virus,

Epstein-Barr virus, cytomegalovirus, pseudorabies virus [5]

C12N 15/39 · · · · · · Poxviridae, e.g. vaccinia virus, variola virus [5]

C12N 15/40 · · · · · Proteins from RNA viruses, e.g. flaviviruses [5]

C12N 15/41 · · · · · · Picornaviridae, e.g. rhinovirus, coxsackie viruses, echoviruses,

enteroviruses [5]

C12N 15/42 · · · · · · · Foot-and-mouth disease virus [5]

C12N 15/43 · · · · · · · Poliovirus [5]

C12N 15/44 · · · · · · Orthomyxoviridae, e.g. influenza virus [5]

C12N 15/45 · · · · · · Paramyxoviridae, e.g. measles virus, mumps virus, Newcastle disease

virus, canine distemper virus, rinderpest virus, respiratory syncytial viruses [5]

C12N 15/46 · · · · · · Reoviridae, e.g. rotavirus, bluetongue virus, Colorado tick fever virus

[5]

C12N 15/47 · · · · · · Rhabdoviridae, e.g. rabies viruses, vesicular stomatitis virus [5]

C12N 15/48 · · · · · · Retroviridae, e.g. bovine leukaemia virus, feline leukaemia virus [5]

C12N 15/49 · · · · · · · Lentiviridae, e.g. immunodeficiency viruses such as HIV, visna-maedi

virus, equine infectious anaemia virus [5]

C12N 15/50 · · · · · · Coronaviridae, e.g. infectious bronchitis virus, transmissible

gastroenteritis virus

C12N 15/51 · · · · · Hepatitis viruses [5]

C12N 15/52 · · · Genes encoding for enzymes or proenzymes [5]

*Note(s) - genes encoding for proenzymes are classified with the corresponding*

*genes encoding enzymes; enzymes are generally categorised according to the*

*"Nomenclature and Classification of Enzymes" of the International*

*Commission on Enzymes. Where appropriate, this designation appears in the*

*groups below in parenthesis.*

C12N 15/53 · · · · Oxidoreductases (1) [5]

C12N 15/54 · · · · Transferases (2) [5]

C12N 15/55 · · · · Hydrolases (3) [5]

C12N 15/56 · · · · · acting on glycosyl compounds (3.2), e.g. amylase, galactosidase,

lysozyme [5]

C12N 15/57 · · · · · acting on peptide bonds (3.4) [5]

C12N 15/58 · · · · · · Plasminogen activators, e.g. urokinase, TPA [5]

C12N 15/59 · · · · · · Chymosin [5]

C12N 15/60 · · · · Lyases (4) [5]

C12N 15/61 · · · · Isomerases (5) [5]

C12N 15/62 · · · DNA sequences coding for fusion proteins [5]

*Note(s)- In this group, the following term is used with the meaning indicated:*

*"fusion" means the fusion of two different proteins. [5]*

C12N 15/63 · · Introduction of foreign genetic material using vectors; Vectors; Use of hosts

therefor; Regulation of expression [5]

C12N 15/64 · · · General methods for preparing the vector, for introducing it into the cell or

for selecting the vector-containing host [5]

C12N 15/65 · · · using markers (enzymes used as markers C12N 15/52) [5]

C12N 15/66 · · · General methods for inserting a gene into a vector to form a recombinant

vector using cleavage and ligation; Use of non-functional linkers or adaptors,

e.g. linkers containing the sequence for a restriction endonuclease [5]

*Note(s) -In this group, the following expression is used with the meaning*

*indicated:*

*"non-functional linkers" means DNA sequences which are used to link DNA*

*sequences and which have no known function of structural gene or regulating*

*function. [5]*

C12N 15/67 · · · General methods for enhancing the expression [5]

C12N 15/68 · · · · Stabilisation of the vector [5]

C12N 15/69 · · · · Increasing the copy number of the vector [5]

C12N 15/70 · · · Vectors or expression systems specially adapted for E. coli [5]

Note(s)This group covers the use of E. coli as host. [5] Shuttle vectors also

replicating in E. coli are classified according to the other host. [5]

C12N 15/71 · · · · Expression systems using regulatory sequences derived from the trpoperon

[5]

C12N 15/72 · · · · Expression systems using regulatory sequences derived from the lacoperon

[5]

C12N 15/73 · · · · Expression systems using phage lambda regulatory sequences [5]

C12N 15/74 · · · Vectors or expression systems specially adapted for prokaryotic hosts other

than E. coli, e.g. Lactobacillus, Micromonospora [5]

*Note(s)This group covers the use of prokaryotes as hosts. [5]*

C12N 15/75 · · · · for Bacillus [5]

C12N 15/76 · · · · for Actinomyces; for Streptomyces [5]

C12N 15/77 · · · · for Corynebacterium; for Brevibacterium [5]

C12N 15/78 · · · · for Pseudomonas [5]

C12N 15/79 · · · Vectors or expression systems specially adapted for eukaryotic hosts [5]

*Note(s)This group covers the use of eukaryotes as hosts. [5]*

C12N 15/80 · · · · for fungi [5]

C12N 15/81 · · · · · for yeasts [5]

C12N 15/82 · · · · for plant cells [5]

C12N 15/83 · · · · · Viral vectors, e.g. cauliflower mosaic virus [5]

C12N 15/84 · · · · · Ti-plasmids [5]

C12N 15/85 · · · · for animal cells [5]

C12N 15/86 · · · · · Viral vectors [5]

C12N 15/861 · · · · · · Adenoviral vectors [7]

C12N 15/863 · · · · · · Poxviral vectors, e.g. vaccinia virus [7]

C12N 15/864 · · · · · · Parvoviral vectors [7]

C12N 15/866 · · · · · · Baculoviral vectors [7]

C12N 15/867 · · · · · · Retroviral vectors [7]

C12N 15/869 · · · · · · Herpesviral vectors [7]

C12N 15/87 · · Introduction of foreign genetic material using processes not otherwise

provided for, e.g. co-transformation [5]

C12N 15/873 · · · Techniques for producing new embryos, e.g. nuclear transfer,

manipulation of totipotent cells or production of chimeric embryos [2010.01]

C12N 15/877 · · · · Techniques for producing new mammalian cloned embryos [2010.01]

C12N 15/88 · · · using micro-encapsulation, e.g. using liposome vesicle [5]

C12N 15/89 · · · using micro-injection [5]

C12N 15/90 · · · Stable introduction of foreign DNA into chromosome [5]

C12Q 1/68 · involving nucleic acids
